# Mapping Gaps in Current Knowledge on the Biology and Ecology of Five Mediterranean Non-Indigenous Fishes: Are International Bibliometric Databases Adequate?

**DOI:** 10.3390/biology15141189

**Published:** 2026-07-18

**Authors:** Francesco Cucinelli, Lorenzo Doria, Gabriele My, Roberta Ingrosso, Timothy Bohan, Francesco Mancini, Giorgio Mancinelli, Paraskevi K. Karachle

**Affiliations:** 1 Department of Biological and Environmental Sciences and Technologies (DiSTeBA), University of Salento, 73100 Lecce, Italy; francesco.cucinelli1@studenti.unisalento.it (F.C.); lorenzo.doria@unisalento.it (L.D.); gabriele.my@studenti.unisalento.it (G.M.); roberta.ingrosso3@studenti.unisalento.it (R.I.); 2 Fondazione DHITECH, High-Tech Technological District, 73100 Lecce, Italy; 3 Bantry Marine Research Station Ltd., Gearhies, Bantry, P75 AX07 Co. Cork, Ireland; timothy.bohan@bmrs.ie; 4 International Centre for Advanced Mediterranean Agronomic Studies (CIHEAM-Bari), 70010 Valenzano, Italy; mancini@iamb.it; 5 National Biodiversity Future Center (NBFC), 90133 Palermo, Italy; 6 Consorzio Nazionale Interuniversitario per le Scienze del Mare (CoNISMa), 00196 Roma, Italy; 7 Institute of Marine Biological Resources and Inland Waters, Hellenic Centre for Marine Research, 19013 Attika, Greece

**Keywords:** biological invasions, lessepsian migrations, non-indigenous species (NIS), invasion dynamics, Mediterranean Sea, bibliometric analysis, gray literature, linguistic barrier, geographic bias

## Abstract

The Mediterranean Sea represents a hotspot of non-indigenous fish species introductions. Here, we provide a comprehensive bibliographic synthesis on five Lessepsian fish invaders—*Fistularia commersonii*, *Lagocephalus sceleratus*, *Pterois miles*, *Siganus luridus*, and *S. rivulatus*—verifying whether international bibliographic databases (IBDs) and online biodiversity information facilities (BIFs) provide adequate information on their occurrence, biology, ecology, and potential or extant commercial use. To this end, we extended to non-indexed bibliographic (NIB) sources available on Google Scholar and compared the results on spatial expansion dynamics and bibliographic trends. We demonstrate that the information provided on occurrence records exclusively by IBD and BIF sources underestimated the total expansion area of invaders by up to 14.8% and determined artifactual temporal voids. Remarkable knowledge gaps were identified in the literature on the reproductive biology and commercial use of all the species, independent of the literature sources, while a relatively larger breadth of information was available on their morphometrics and trophic ecology. Noticeably, NIB sources provided more complete bio-ecological information, particularly for *L. sceleratus*. In addition, the majority of the studies on commercial uses were published in NIB sources, to the point that for some species, IBD sources provided negligible or null information on the topic. This study indicates that disregarding non-indexed literature sources might exacerbate knowledge voids exclusively related to linguistic barriers or geographic biases, weakening the scientific support necessary for identifying appropriate strategies of control and mitigation of bioinvasions in the Mediterranean Sea.

## Simple Summary

The Mediterranean Sea is increasingly affected by fish species migrating from other regions, particularly from the Red Sea. Managing these bio-invasions requires a plethora of reliable scientific information; however, not all available knowledge is accessible through major scientific journals. Here, we examined the literature available on five invasive fish species (*Fistularia commersonii*, *Lagocephalus sceleratus*, *Pterois miles*, *Siganus luridus*, and *S. rivulatus*) and compared the data found in major international scientific databases and online biodiversity platforms with the information from non-indexed, less visible sources, including local journals, reports and documents available from Google Scholar. We found that relying only on international databases can track the general trend of spatial expansion, but underestimates the geographical spread of invasive species, creating false gaps in historical records and their expansion. Important missing information was also detected for major topics across both indexed and non-indexed sources, like reproduction and commercial uses of these fish, while their dietary habits and morphometrics were more frequently studied. Non-indexed publications often provided more detailed ecological and biological information and contained most of the available studies on commercial exploitation. Our results showed that the exclusion of non-indexed literature can produce geographic bias and linguistic barriers, ultimately limiting the breadth of knowledge available to researchers and decision-makers for implementing adaptive strategies to bioinvasions.

## 1. Introduction

Biological invasions are one of the major anthropogenic pressures on natural ecosystems worldwide, significantly threatening biodiversity, ecosystem functioning and services at different organizational levels [1]. In particular, the Mediterranean Sea has been under siege by non-indigenous species (NIS) and Invasive Alien Species (IAS), with more than 1000 introductions to date [2], with the major pathway of NIS being the Suez Canal, a process known as *Lessepsian migration*, with more than 600 Lessepsian species currently reported [3,4,5]. Over the past few decades, the phenomenon has intensified in frequency and extent, driven by climate change—especially for thermophilic species and within the Eastern Mediterranean—as well as by increased shipping traffic and habitat alterations favouring rapid population expansions by NIS, thus profoundly altering recipient communities and ecosystems [6,7,8].

Mitigating the impacts of established NIS is a fundamental strategy recognized by the European Commission for achieving Good Environmental Status (GES), by both preventing new NIS introductions, range expansions and establishment along the European coasts and keeping established NIS populations at levels that “do not adversely alter the ecosystems”, as expressed in the Marine Strategy Framework Directive (MSFD) [9]. However, while in terrestrial and enclosed water systems several NIS have been successfully managed, in marine environments, NIS management and control are particularly challenging due to the greater difficulty in early detection and to the high connectivity of marine environments, greatly favouring the recolonization, especially for NIS, with high dispersal capabilities [10,11].

Management of IAS requires detailed and comprehensive knowledge of the species biology (e.g., size, age, sex structure, growth and mortality, reproduction and recruitment) and ecology (e.g., abundance, feeding habits, trophic level, competitive interactions with native species), towards performing an informed stock assessment and to assess the actual ecological and socio-economic impacts of these species, since this information is usually lacking outside of the species native ranges [12,13,14,15,16,17]. This information is crucial when trying to develop targeted fisheries for these species, as highlighted by the FAO-GFCM with the establishment of the “Recommendation GFCM/42/2018/7” on blue crabs [*Callinectes sapidus* (Rathbun, 1896) and *Portunus segnis* (Forskål, 1775)], aiming at the collection of context-specific information on the species biology and ecology across the Mediterranean and development of fishery management actions [18]. Moreover, limited data are currently available on these species’ commercial uses—as most NIS are poorly studied in terms of nutritional values, biochemical composition, market acceptance, and potential industrial applications—despite the growing interest in turning NIS into potential economic opportunities, with few exceptions being represented by iconic species (e.g., the blue crab *C. sapidus* and the lionfish *Pterois* spp.).

The robust assessment of the distribution of NIS, together with the characterization of their biological and ecological traits within invaded ranges, remains a global challenge [19]. Efforts in collecting such information on NIS are frequently hindered by linguistic barriers and systemic geographic bias in academic publishing—a bias that extends beyond linguistic issues alone and mainly originates from the exclusion of regional, non-indexed journals that publish in English but remain absent from major international databases. Such exclusions can lead to a distorted knowledge landscape for non-Anglophone or developing regions and a fragmented understanding of global invasions [20,21,22,23]. The integration of indexed and non-indexed literature sources is therefore essential to mitigate such incongruences, as non-indexed sources often represent the only repository of small-scale and regional studies [23,24]. Traditional bibliometric databases, such as Scopus and Web of Science (WoS), represent the gold standard for scholarly indexing; however, their selective inclusion criteria tend to prioritize journals with higher citation metrics and international reach, potentially underrepresenting regional and non-Anglophone outputs. While international bibliographic databases offer highly structured and standardized data, a substantial amount of critical information remains external to these platforms and is identified as “non-indexed literature”. Non-indexed literature encompasses a wide, heterogeneous spectrum of sources with different degrees of formal quality, spanning from peer-reviewed articles published in regional journals lacking international indexing to “gray literature”—such as academic theses, conference proceedings and posters, institutional technical documents and non-peer-reviewed reports [23,25]. In contrast, Google Scholar provides a more general “superset” of publications that has proven to consistently retrieve a significantly wider spectrum of the literature across disciplines [26], making it a valuable tool to intercept non-indexed literature. Adopting more expansive search strategies is particularly important in invasion biology, where non-indexed literature may represent a critical supplement of knowledge for impact assessment and management, while also helping in reducing biases associated with the reliance on indexed sources alone [27].

In recent years, research has often focused on mitigating the costs of NIS management by the integration of these species into local economies, through the promotion of their commercial applications or uses—as alimentary or non-alimentary products—as well as recreational utilization, thus turning otherwise harmful species into economic opportunities, which can create a win–win solution for NIS management [28,29,30]. Usually, the main use attributed to invasive species is that of an alimentary product, with several instances worldwide—e.g., the lionfish *Pterois* spp. in the south-east Atlantic region and the blue crab *C. sapidus* in the Mediterranean Sea [28,31]. Additionally, efforts are currently being made to develop the utilization of NIS in other industries, such as pharmaceuticals and cosmetics.

Here, we focused on five prominent Lessepsian fish species: the bluespotted cornetfish (*Fistularia commersonii* Rüppell, 1838), the silver-cheeked toadfish (*Lagocephalus sceleratus* Gmelin, 1789), the common lionfish (*Pterois miles* Bennett, 1828), the dusky spinefoot (*Siganus luridus* Rüppell, 1829), and the marbled spinefoot (*Siganus rivulatus* Forsskål & Niebuhr, 1775). The species were selected within the VALIAS project, based on their invasion status, abundance and existing knowledge, to be explored for potential use in the food, feed and cosmetics industry. The primary aim of our investigation was to evaluate whether the inclusion of non-indexed sources alters our understanding of the distribution, invasion dynamics, ecological knowledge, and economic valorization of invasive fishes in the Mediterranean. To address it, we integrated information from Indexed Bibliographic Databases (IBDs), online Biodiversity Information Facilities (BIFs), and non-indexed bibliographic (NIB) sources. Specifically we aimed to: (1) assess the contribution of NIB sources in reconstructing species spatio-temporal expansion patterns and occurrence records; (2) evaluate the temporal relationship between invasion dynamics and scientific attention; (3) identify potential knowledge gaps in the overall literature and evaluate whether the inclusion of NIB sources can improve in our understanding of these species biology, ecology and potential commercial uses; and (4) characterize geographical, linguistic and collaborative biases in the available knowledge. Through these complementary analyses, we tested whether indexed sources alone (i.e., IBDs and BIFs) can provide a sufficiently comprehensive representation of Mediterranean bioinvaders.

## 2. Materials and Methods

### 2.1. Literature Search and Data Entry

The available literature sources on the five target species were screened in agreement with the PRISMA (Preferred Reporting Items for Systematic Review and Meta-analyses) guidelines [32,33] using Google Scholar^®^. We did not perform the search using other international databases, such as Scopus or WoS, because the aim was to collect both IBD and NIB sources as well as non-Anglophone publications or publications in regional journals and gray literature. The details of the search procedure and its results are provided in Appendix A and Table A1. In brief, the initial structure of the query was: (“scientific name” OR “common name”) AND (“Mediterranean Sea” OR “invaded range”). Further relevant publications were identified by inspecting the references for the retrieved sources. Once the initial data collection and entry were completed, additional topic-specific queries were performed, adding a third set of keywords [e.g., AND (“occurrences”); AND (“length–weight relationship” OR “growth”), etc.], based on the thematic categories formalized in the database [i.e., length–weight relationships (LWRs), growth parameters, spawning periods and fecundity, diet composition and food items, and possible commercial uses]. To validate the comprehensiveness of the retrieval via Google Scholar and to formally differentiate between IBD and NIB sources, each query was cross-validated in both Scopus and WoS, confirming that the 100% of the literature available in IBD was successfully retrieved by the primary search in Google Scholar.

All the collected information regarding the biology of the species was entered in a multi-sheet database, formalized using FishBase [34] tables of each topic as a reference, duplicating records for multiple entries from the same paper. The “references” sheet was employed for storing the bibliographic citations metadata in 10 columns, including a unique identifier (reference_id), the valid taxonomic name of the species, the publication year, authors (for more than two authors, the abbreviation “et al.” was used), title, topic, DOI, and their alien status (y/n). Each reference was duplicated when a paper addressed multiple topics and/or species. Finally, reference_id codes were used as a key in all other worksheets to link each entry to the original source.

### 2.2. Occurrence Data

Occurrence records (simply occurrences from now on) obtained from bibliographic sources were complemented with information from data papers and online databases [35,36,37,38,39] as well as from BIFs (i.e., the Global Biodiversity Information Facility [GBIF, www.gbif.org, accessed on: 30 August 2025] and the Ocean Biogeographic Information System [OBIS, www.obis.org, accessed on: 30 August 2025]), following the procedure described elsewhere [40,41,42,43,44,45]. The literature search extended to 1 August 2025. Geographical distribution data were stored in the “geographic_references” sheet (22 columns), recording precise and approximated locations and following standardized marine regionalization in agreement with the MSFD [9], as modified by Zenetos et al. 2010 [3]. Data include the species group, marine region and subregion, locality, coordinates, sampling date, depth range, abundance and substrate type. For occurrences with no coordinates, latitude and longitude were derived from Google Earth based on geographical information reported in the original source (e.g., distance from reference landmarks like cities, beaches, ports or the centroid of bays and gulfs) [39,40]. In addition, more complex multi-location and multi-year samplings were entered as multiple rows.

### 2.3. Data Analysis

All data analyses were performed in the R Software (v. 4.5.1) [46], using the *ggplot2*, *ggraph* and *circlize* packages for data visualization [47,48,49] and the *dplyr* package for data manipulation [50]. Statistical significance was evaluated at α = 0.05.

Occurrence data were aggregated and preliminarily checked to exclude entries for preserved specimens and those with missing or insufficient decimal precision of the coordinates. Finally, occurrences were filtered for duplicates within the same year and a distance range of 50 m and successively mapped using the *sf* package [51]. Cumulative spatial expansion curves were explored following refs. [52,53]. A segmented linear regression model was applied to the cumulative number of new occupied cells in a reference EEA grid of 10 × 10 km (available at https://sdi.eea.europa.eu/data/e834751f-19d1-4842-823d-e90e600c5993, accessed on 28 February 2026) using the *segmented* package [54], allowing the identification of two breakpoints (BPs) in the cumulative curves and of slope variations across three time periods, corresponding to the arrival (or lag-phase), establishment (or dispersal) and expansion or saturation phases, in concordance with the classical invasion dynamics literature [55,56,57,58,59]. We used an identical approach to analyze the cumulative curve of the number of publications, allowing the identification of breakpoints and the rate of response in the scientific literature.

To evaluate whether excluding the non-indexed literature results in differences in species occurrence patterns and expansion dynamics, the temporal trends of cumulative new occupied cells between indexed-only sources—thus excluding NIB sources—and total sources were compared, computing the number of excluded cells at the final point of the cumulative curves (i.e., 2025) and, on average, along the whole time of each species invasion history. Additionally, divergence between the two curves was measured by using the root mean square deviation (RMSD), computed as the square root of the mean squared differences between the standardized cumulative curves. Generalized Least Squares (GLS) models with a first-order autoregressive correlation structure (AR1), fitted using the *nlme* package [60], were employed to compare the relation between the invasion dynamics and the attention given by the scientific community for each species, only considering the total sources. This approach verified whether increases in invasion extents were associated with synchronous increases in scientific attention, explicitly modeling for temporal autocorrelation of the data. Prior to modeling, both variables were standardized through Z-scores to remove differences in scale and allow direct comparison of effect sizes. The GLS models used the standardized annual increment of publications as the response variable, with the standardized annual increment in occupied cells as the predictor. In both cases, models were fitted via restricted maximum likelihood (REML), and the strength and direction of the relationship were inferred from the regression coefficient (β), while the autoregressive parameter (φ) was used to quantify residual temporal autocorrelation.

The collected bibliographic sources were grouped according to their scientific topics, defined in three major macro-categories: (1) biology, encompassing the sub-categories length–weight relationships, growth (including the von Bertalanffy parameters of growth functions, mortalities, and length and age at maturity) and reproduction (including absolute fecundity, relative fecundity and spawning season); (2) trophic ecology and feeding habits (including studies on the diet composition, both in qualitative and quantitative terms, from stomach content analyses); and (3) commercial uses (including extant or potential commercial or industrial uses and/or applications across multiple sectors, such as pharmaceutical, medical, biomonitoring, cosmetic, and alimentary safety, as well as the evaluation of meat chemical composition and ornamental trade). Cumulative growth curves were subsequently computed per macro-thematic area to identify shifts in research trajectories over time for both indexed-only and total sources. To perform the bibliometric analysis, all bibliographic sources were retrospectively cross-referenced in WoS and in Scopus to compile a comprehensive metadata repository of all the references cited therein. Further, NIB sources were manually formatted into additional BibTeX files using citation data retrieved directly from Google Scholar. The consolidated database was subsequently cleaned of duplicate entries and analyzed using the *bibliometrix* R package [61]. Keywords co-occurrence networks were constructed for each species based on association strength, employing the Leiden clustering algorithm to identify distinct thematic communities, and were finally visualized using the Fruchterman–Reingold force-directed layout, after an initial standardization of the keywords against a standardized vocabulary. Finally, *bibliometrix* was used to build an international collaboration network based on the institutional affiliations of the authors. This network utilized Jaccard’s similarity index to map the geographic distribution and connectivity of research efforts. The analysis was finally complemented by an assessment of the temporal trends of research contributions from different countries.

## 3. Results

### 3.1. Species Distribution and Expansion Patterns

**Figure 1:**
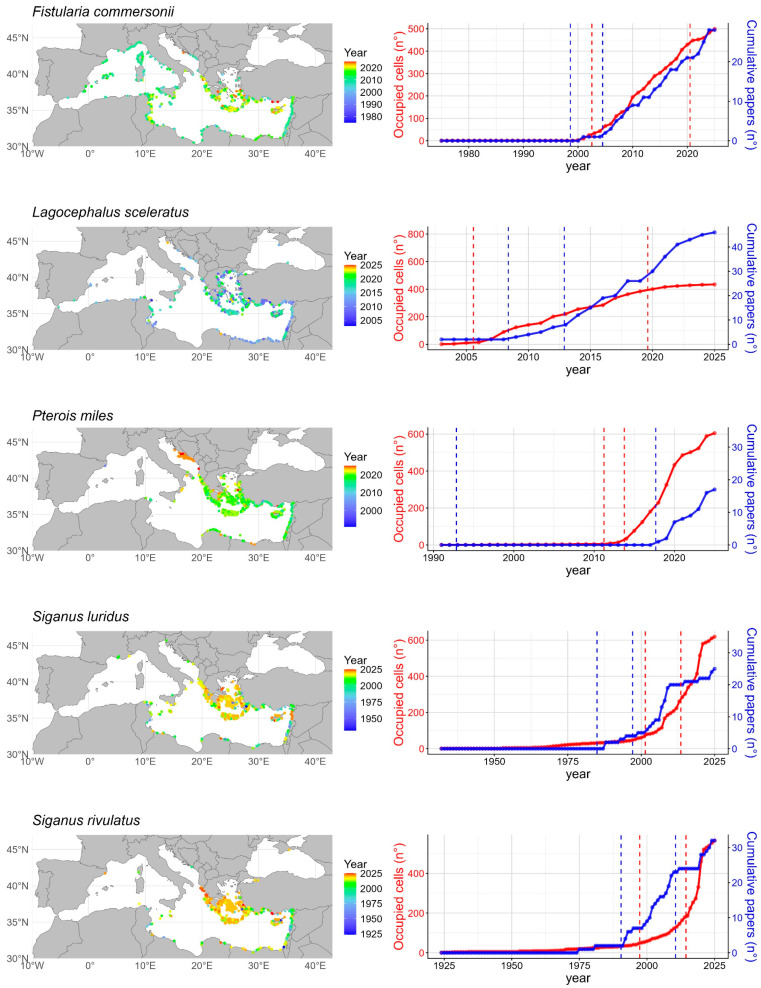
(**Left**) Distribution maps of the five target species within the Mediterranean Sea; (**right**) comparison of the cumulative curves of the number of new occupied cells and of the number of bibliography sources per year; blue and red vertical lines represent the curves computed breakpoints.

Occurrences extraction from the literature and online databases and their successive selection allowed the identification of more than 16,000 occurrences worldwide and 7340 in the Mediterranean Sea for all five species. Table 1 summarizes the number of Mediterranean occurrences of each species, along with the information on their first registered Mediterranean occurrence. *L. sceleratus* exhibited the lowest number of Mediterranean occurrences, while the two siganids dominated the Mediterranean dataset (2099 and 1678 occurrences, respectively). The oldest occurrence was for *S. rivulatus* in 1924 (Palestine), while the most recent was for *L. sceleratus* in 2003 (Turkey). In Mediterranean waters, occurrences clustered predominantly along the Levantine and Aegean coasts and extended westward, reaching the Spanish and Algerine coasts of the Mediterranean Sea (Figure 1). Approximately 50% of occurrences from the Mediterranean were collected from BIFs for all species. The remaining occurrences were predominantly retrieved from IBD sources, ranging from a minimum of 32.7% in *F. commersonii* to a maximum of 46.7% in *P. miles*, and to a minor extent from NIB sources, with contributions ranging from 3.2% in *S. rivulatus* and 13.8% in *F. commersonii*. Interestingly, the species with the lowest overall occurrences within the Mediterranean (i.e., *F. commersonii* and *L. sceleratus*) showed the highest percentage of occurrences from NIB sources, doubling those of all the other three species combined.

### 3.2. Invasion Trajectories and Cumulative Curves

**Figure 2:**
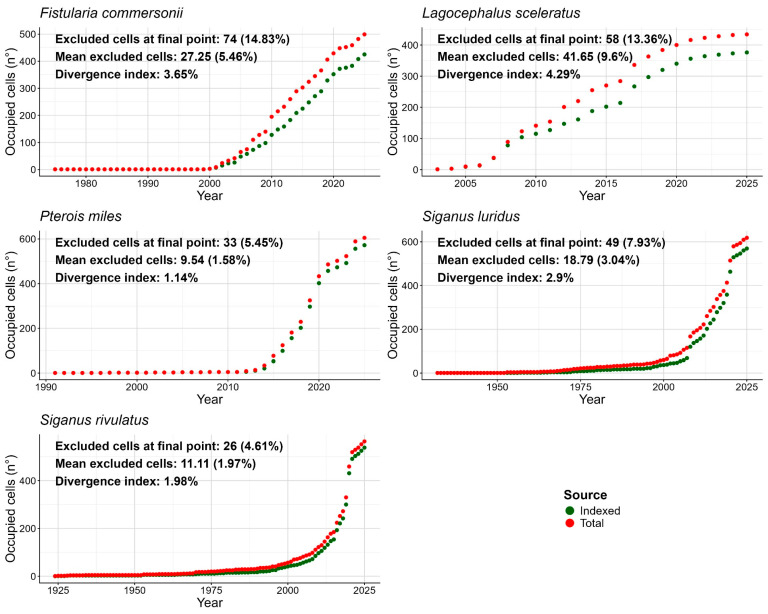
Comparison of cumulative curves of newly occupied cells for the total and indexed-only dataset. Number and percentage of excluded cells are presented for the final time point and on average, along with an overall divergence index based on root mean square divergence (RMSD).

The number of occupied cells in the 10 × 10 km grid ranged between 434 (*L. sceleratus*) and 618 (*S. luridus*). When compared, the cumulative curves of newly occupied cells for the total sources and the indexed-only sources highlighted similar trends, with a divergence ranging between 1.14% for *P. miles* and 4.29% for *L. sceleratus* (Figure 2). Considering the entire timespan of invasion, the mean number of excluded cells followed the same pattern of the divergence index, peaking at 9.6% (41.65 cells) for *L. sceleratus*. However, the final number of excluded cells at the last time point was over the double of the mean value for all species except *L. sceleratus*, ranging from a minimum of 26 cells (4.61%) for *S. rivulatus* and a maximum of 74 cells (14.83%) in *F. commersonii* (Figure 2).

The cumulative spatial expansion curves (Figure 1) showed different invasion trajectories across species, all characterized by breakpoints, ultimately confirming significant changes in invasion rates. Most species followed a three-phasic model (Table 2). *F. commersonii* and *L. sceleratus* showed a sigmoidal curve (*sensu* Hui et al. (2017) [59]), characterized by an initial lag, followed by a steep acceleration and a final saturation phase (after 2021 and 2020, respectively). Notably, *L. sceleratus* displayed the most atypical curve, experiencing the shortest arrival phase (3 years) and a prolonged expansion phase until saturation. Conversely, *S. luridus* and *S. rivulatus* followed the typical J-shaped exponential trend, with a marked acceleration after 2013 and 2014. *P. miles* represented the only biphasic exception, as the slopes of phases 1 and 2 did not differ significantly (*p* = 0.87). Even though they showed different trends, both *P. miles* and *F. commersonii* remained virtually undetected for over 20 years since their first occurrences, experiencing burst accelerations after 2014 and 2003 (Δ = +51.52 and Δ = +23.63, respectively).

### 3.3. Trends in Literature Efforts

A total of 110 unique literature sources were selected for the five target species, focusing on length–weight relationships (LWR), growth parameters, mortality, fecundity, spawning, diet and potential commercial uses, and excluding those sources providing only occurrences. In particular, the number of sources for *F. commersonii*, *L. sceleratus*, *P. miles*, *S. luridus*, and *S. rivulatus* was 29, 46, 17, 26, and 33, respectively. Noticeably, between 23 and 57% of the identified literature sources were not indexed in IBD (Table 3), with *L. sceleratus* being mostly represented in NIB sources.

The trends in the accumulation of the literature showed remarkable interspecific differences (Figure 1). Specifically, for *F. commersonii*, the growth in the attention of the scientific community paralleled the expansion of the species, with a maximum lag of only two years, while for *P. miles* and *L. sceleratus* publications lagged the respective expansion phases by 4–5 years (Table 4). Conversely, for both siganids, the accumulation of publications preceded their spatial expansion by nine years for *S. luridus*, and 14 years for *S. rivulatus* (Figure 1; Table 4). The pair experienced a deceleration of accumulation after 2010 (Δ = −6.25 for *S. rivulatus*), with almost no publications recorded until 2020 for *S. rivulatus* and 2023 for *S. luridus*—although no significant breakpoint was identified by the segmented model for the latter. GLS models showed a low to moderate temporal autocorrelation, with φ values ranging from 0.13 for *F. commersonii* to 0.47 for *P. miles*. The models also confirmed a significant synchrony between the rate of spatial expansion and the accumulation of the literature for most of the species (Table 4). In particular, *P. miles* showed the highest β coefficient (0.891), followed by *S. rivulatus* and *F. commersonii*. Conversely, for *L. sceleratus* and *S. luridus*, non-significant relationships were observed (Table 4).

### 3.4. Bibliometric Patterns

#### 3.4.1. Research Effort Across Species and Topics

**Figure 3:**
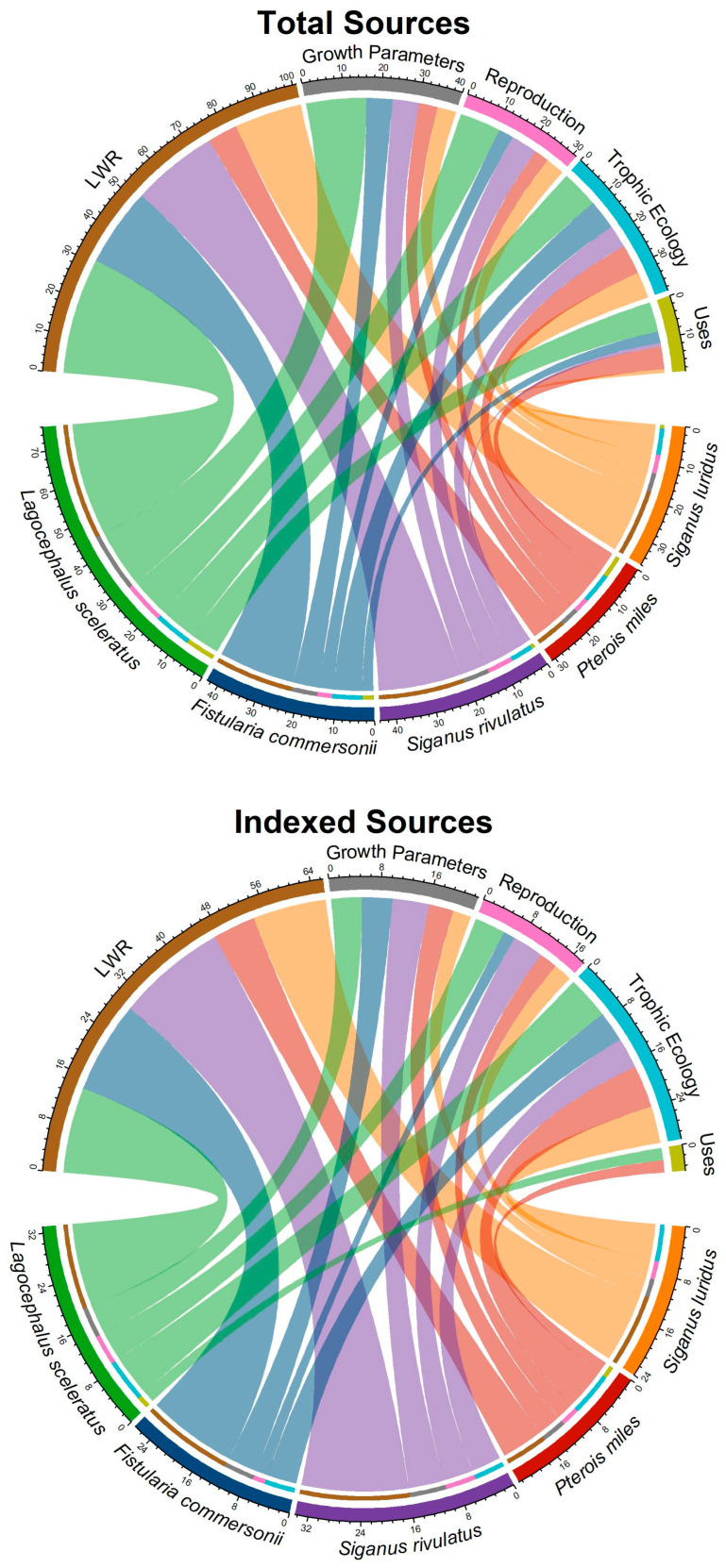
Chord flow-charts of the number of sources per species and per research topic, for total sources (**up**) and indexed sources only (**down**). Sources covering more than one topic are duplicated to better express research attention on each topic. Because of the scarcity of data on spawning and fecundity of the species, the two categories were collapsed into a general “reproduction” category.

Similar patterns in the number of sources per topic were observed for both total and indexed-only sources (Figure 3), with “LWR” and “Uses” covering the highest and lowest proportion of sources, respectively, while the three remaining topics show similar proportions in both total and indexed-only sources. “LWR” was the best represented topic (105 sources), while “Uses” (19) and “Fecundity” (14) were the least represented topics. *Lagocephalus sceleratus* was the species dominating each research topic, covering between 25% (Trophic Ecology) and 50% (Uses) of sources in each category. Conversely, *P. miles* represented the species with the lowest number of publications in each research topic, with the only exception being the reported commercial uses. The two siganids and *F. commersonii* generally showed a similar behavior across categories (Figure 3). In addition, research efforts on commercial uses were fundamentally focused on potential and extant alimentary and pharmaceutical applications of the species, with *L. sceleratus* showing the highest diversity in commercial uses and the siganids showing the lowest diversity (only one reported alimentary use). Lastly, fecundity sources were the least abundant category (14 sources), with a minimum of only 1 source in *P. miles*, and spawning-only for 27 sources, peaking for *L. sceleratus* (n = 11, 40.7%). On the other hand, when considering only the indexed sources, species showed similar proportions in all categories, with the sole exception of *P. miles* in the topic “LWR”, and *P. miles* and *F. commersonii* in the topic “Reproduction”. Interestingly, the siganids and *F. commersonii* had no reported commercial uses in indexed sources.

#### 3.4.2. Temporal Accumulation Dynamics of Research Topics

**Figure 4:**
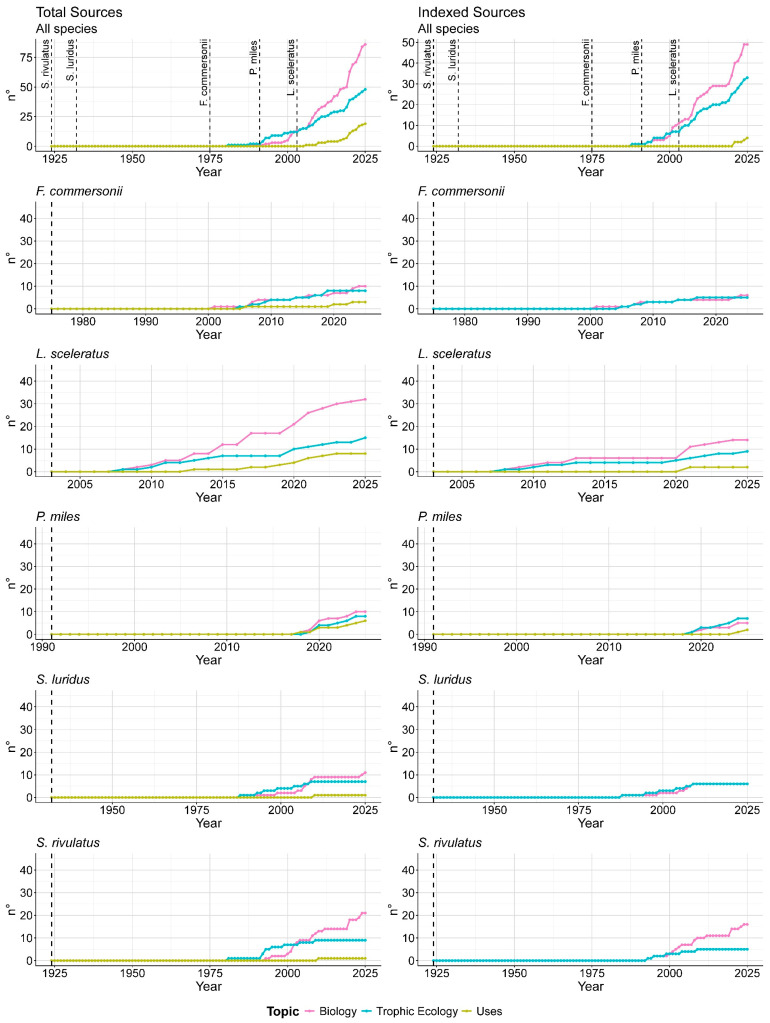
Comparison of cumulative curves of the number of sources between total and indexed-only sources for all species (**up**) and single species (**down**). Black vertical lines indicate the year of first introduction. Sources covering more than one topic are duplicated to better express the research attention on each topic.

Cumulative curves were also computed, differentiating the sources in three research topics, namely “Biology” (including LWR, growth and reproduction), “Trophic Ecology” and “Uses” for both total and indexed-only sources (Figure 4). For the total sources, the three topics were characterized by different growth patterns, with the biology and commercial uses curves expressing exponential growth and the trophic ecology growing linearly. Also, trophic ecology was the first topic to start growing, since the early 1980s, while “Biology” exploded after 2000 and commercial uses only in the last decade to 15 years. Separating the topics in each species with distinct research trajectories showed that *F. commersonii* displays perfect synchrony and a linear growth in studies on its biology and trophic ecology, while the contribution of investigations on commercial uses was limited and started only after 2020. The siganids showed no growth in their potential commercial uses and an exponential growth in studies regarding biology, while studies on their trophic ecology showed a sigmoidal growth and a flattening after the early 2000s. On the other hand, *L. sceleratus* showed a steady and almost synchronized linear growth across categories, although sources on commercial uses increased with a lag of approximately four years with respect to the other two categories (Figure 4). Finally, *P. miles* displayed a quite compressed research history, with all three topics emerging simultaneously in the last ten years and showing an exponential and similar growth across categories. The comparison of total and indexed-only sources revealed a time lag in the overall reporting of commercial uses, with indexed sources appearing only after 2021. In contrast, biology and trophic ecology showed a similar, although slower, growth trend; notably, biology was characterized by a huge gap in indexed publications between 2014 and 2018. At the species level, indexed sources accumulation dynamics resulted consistently slower, although no lag was detected for biology and trophic ecology topics across most species, with the exception of the lionfish, which showed a 4-year delay in biological studies (Figure 4). On the other hand, studies on commercial uses were absent for *F. commersonii* and the siganids, while *L. sceleratus* and *P. miles* exhibit lag times of eight and four years, respectively. For *F. commersonii* and *S. rivulatus*, indexed sources showed similar trends to total sources in both trophic ecology and biology. Conversely, while trophic ecology trends remained consistent for the remaining species, a major gap was detected in *L. sceleratus* between 2013 and 2020 in indexed biology studies.

### 3.5. Research Landscape and Knowledge Mapping

**Figure 5:**
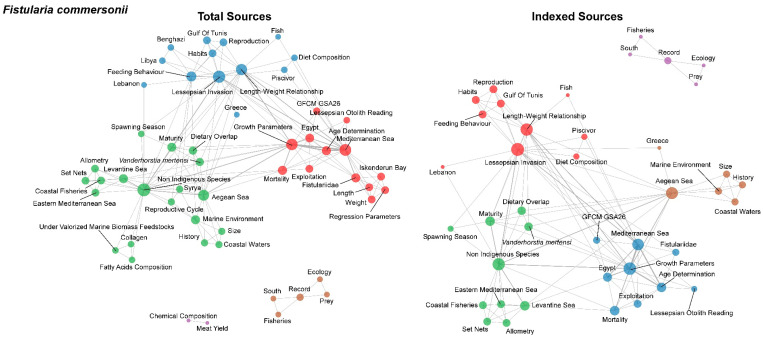
Co-occurrence network for keywords for *Fistularia commersonii* literature sources for total sources (**left**) and indexed-only sources (**right**). Node colors distinguish different clusters of keywords based on Leiden clustering.

**Figure 6:**
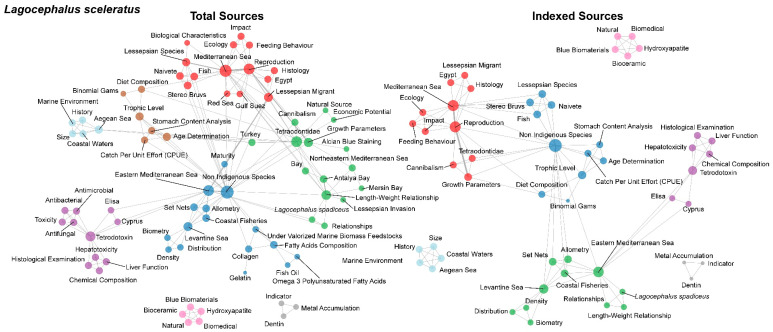
Co-occurrence network for keywords for *Lagocephalus sceleratus* literature sources for total sources (**left**) and indexed-only sources (**right**). Node colors distinguish different clusters of keywords based on Leiden clustering.

**Figure 7:**
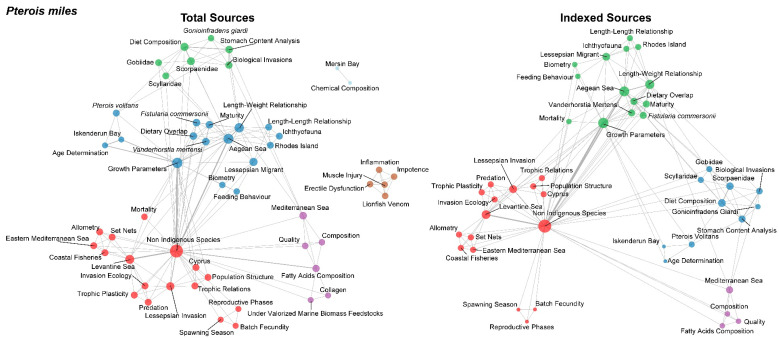
Co-occurrence network for keywords for *Pterois miles* literature sources for total sources (**left**) and indexed-only sources (**right**). Node colors distinguish different clusters of keywords based on Leiden clustering.

**Figure 8:**
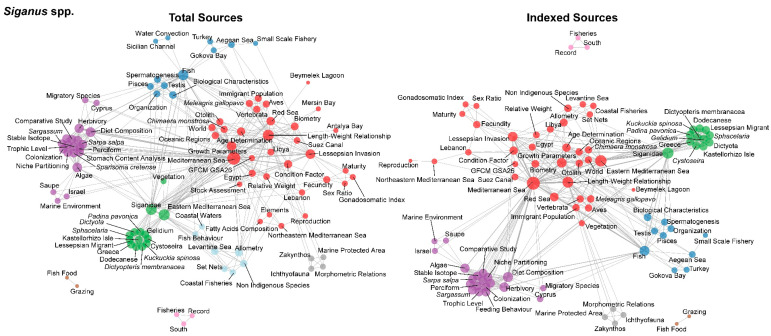
Co-occurrence network for keywords for *Siganus luridus* and *S. rivulatus* literature sources for total sources (**left**) and indexed-only sources (**right**). Node colors distinguish different clusters of keywords based on Leiden clustering.

The collection of bibliographic metadata allowed the construction of keywords co-occurrence networks for all species, considering both the total sources and the indexed-only sources (Figure 5, Figure 6, Figure 7 and Figure 8). The comparative analysis of networks (Table 5) revealed a discrepancy in the structural properties of the keyword co-occurrence networks—specifically, the number of nodes and the density—shifted markedly when restricting the analysis to indexed sources alone. Across all species, the number of nodes (keywords) consistently decreased in the indexed sources-only networks, indicating the reduction in the thematic breadth of the research. Conversely, the network’s density was higher for the indexed-only dataset compared to total sources, highlighting a tighter, more standardized core of co-occurrences in the indexed sources.

In the networks derived from total sources, the number of interconnected clusters ranged from a minimum of three for *F. commersonii* to a maximum of six for the siganids. In all cases, isolated clusters completely detached from the primary network were observed. Indexed-only sources generated networks with a similar structure, although simplified by the disappearance of nodes and clusters. The networks’ core across species was typically composed of three clusters (identified in red, blue and green in Figure 8), remaining largely unaltered in the shift to indexed networks and dominated by keywords strictly related to the species biology and ecology. On the other hand, peripheral clusters often disappeared in indexed-only networks and were dominated by keywords related to fisheries and commercial uses. Furthermore, geographical granularity was significantly higher for total sources, with keywords identifying geographical localization of the studies (e.g., Benghazi, Libya, Iskenderun Bay), typically disappearing after removal of non-indexed sources. Interestingly, both *L. sceleratus* and *P. miles* networks (Figure 6 and Figure 7) were characterized by investigations focusing on their toxicity and venomousness, although in *P. miles*, the major cluster on the topic was represented by a single non-indexed paper. In contrast, siganids maintained a high degree of complexity in both networks, with a clear separation of trophic ecology thematics (identified in green and purple) from biological studies (identified in red and blue) (Figure 8).

**Figure 9:**
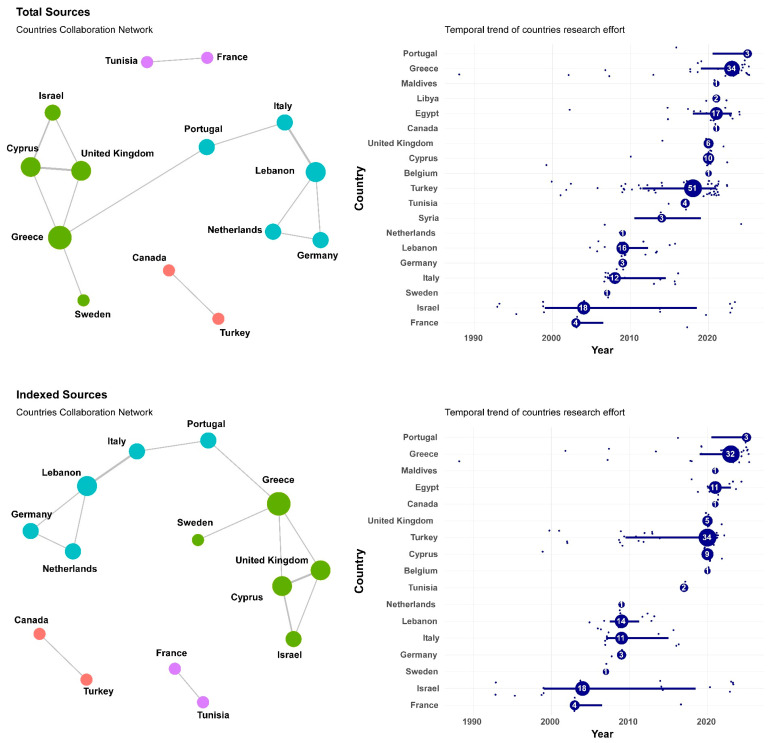
Collaboration network and trend plot of countries’ research effort across the five species for total literature sources (**up**) and indexed-only literature sources (**down**). Node size is proportional to a country’s degree of centrality (i.e., the total number of international links), while edge thickness represents the strength of each bilateral cooperation between countries normalized by the Jaccard index. Node colors distinguish different clusters of collaborating countries based on Leiden clustering. Numbers in the trend plots indicate the number of research papers by country.

The collaboration network (Figure 9) remained topologically stable after removal of non-indexed sources, although the strength of connections weakened. Two primary clusters emerged: a large “Levantine” group—excluding Turkey and Lebanon and bridged with the UK and Sweden—and a “European” group, including Lebanon. Isolated dyads were formed by Turkey–Canada and France–Tunisia, while countries like Libya, Egypt and Syria had no connections and were excluded from the network. Temporal trends in research effort (Figure 9) followed three identical phases across both datasets: (1) a “pioneering phase” led by France and Israel until the early 2000s; (2) an “expansion phase” post-2005 with rising contributions from Italy, Cyprus, Lebanon and Turkey; and (3) the current phase, post-2020, dominated by Greece, Turkey and Egypt. However, the exclusion of non-indexed sources disproportionately obscured the research effort of many Levantine and North African countries: for example, Turkey and Egypt lost 17 and 6 sources, respectively, while Syria and Libya disappeared from scientific records.

## 4. Discussion

### 4.1. Species Distribution and Occurrence

The current investigation provides a comprehensive and updated overview of the distribution patterns and invasion history for five Lessepsian fish species within the Mediterranean. The occurrences, distributions and trends for the five species (Figure 1) confirmed the classic north- and westward expansion trends for Lessepsian species with pelagic propagules [62,63]. Occurrences are largely concentrated in the Eastern Mediterranean area and the Ionian Sea, with *P. miles* representing the only species not yet established in the Western Mediterranean, and *F. commersonii* being the most widespread species in this area (Figure 1). Overall, this pattern is consistent with a stepping-stone progression interrupted by circulation dynamics and thermal barriers between Mediterranean sub-basins. However, it is worth noting that most of the new occurrences after 2020 were from the Eastern Mediterranean area, with only a few occurrences of *F. commersonii* and *L. sceleratus* in the Adriatic and Western Mediterranean and several occurrences of *P. miles* in the Eastern Adriatic Sea.

This study confirms the pivotal role of online BIFs (i.e., GBIF and OBIS) in mapping species distribution and invasion history and highlights how the use of IBD sources only is an effective and robust approach for tracking species’ broad temporal expansion trends (i.e., there was almost negligible divergence in Figure 2). However, we acknowledge that the exclusion of NIB sources can cause the underestimation of the overall area of expansion up to 14.8% and could introduce temporal and geographical voids in species expansions. In particular, while cumulative expansion curves for *P. miles* and *Siganus* spp. showed classic exponential growth, *F. commersonii* and *L. sceleratus* were characterized by more articulated cumulative expansion curves, suggesting that these species might have reached their maximum potential expansions within the Mediterranean under current environmental conditions. Both species are among the most rapidly expanding Lessepsian invaders and have already reached the northernmost and westernmost limits of the Mediterranean basin, which is consistent with a near-saturation colonization [64,65]. Nevertheless, this interpretation should be treated with caution, as the observed patterns may also reflect spatial heterogeneity in sampling effort and reporting intensity across the basin.

Lastly, *L. sceleratus* showed a particularly short arrival phase compared to all other species (three years), with a burst explosion in occurrences soon after its arrival. This might indicate that the species was not adequately reported and thus recorded upon arrival (Table 2; Figure 1). Such delays in recording and reporting of new NIS can be frequent, particularly during their arrival stage and for Lessepsian migrants, as highlighted by the lag observed for *F. commersonii* between its first occurrence in 1975 and its actual confirmation in the scientific literature only in 2014 and for the 21-year time lapse for *P. miles* [56,66,67,68,69,70]. However, it must be acknowledged that our interpretation of cumulative curves must be carefully considered, as occurrences do not necessarily represent actual species distributions, but rather the human perception of species distribution and density. Indeed, the global boom in citizen science campaigns over the last decade, alongside increased database mobilization and reporting practices, has significantly accelerated the accumulation of occurrences. This is particularly true for species with distinctive morphologies in the Mediterranean, such as those considered in this study. This includes several Mediterranean initiatives, such as the Italians’ “AlienFish” and “Attenti a quei 4!” (2022; CNR-IRBIM and ISPRA)—targeting non-indigenous and rare fish species; the Greek and Cypriot projects “RELIONMED-LIFE”—specifically focused on lionfish detection and culling; and “Is it alien to you? Share it!” (iSea and MER Lab). Therefore, the apparent “burst” in occurrences for certain species over others might have been determined by the synergistic effect of both biological expansion and increased observation effort.

### 4.2. Research Effort and Bibliometric Patterns

Spatial expansion explained the literature accumulation trend for three of the five species, but not for *L. sceleratus* and *S. luridus* (Table 4). Regarding the latter, the lack of correlation between the literature sources and spatial expansion cumulative curves is related to the combined effect of both a gap in publication experienced after 2010 and to the overall low number of literature sources available (25) relative to its final spatial range (608 cells) (Figure 1). Conversely, *L. sceleratus* experienced a disproportionate accumulation of literature sources with respect to its spatial expansion, highlighting how publication output is strongly modulated by a combination of public health risks, socio-economic interests, and targeted funding rather than mere spatial expansion. This species is perceived as a high-impact species within the Mediterranean because of its interference with fishing gears and human health concerns—related to both its high tetrodotoxin content and physical attacks [71,72]. Therefore, policy concerns have likely triggered targeted research funding and dedicated monitoring initiatives, confirming the urge for research on this species. The urge for research on the species is confirmed, as *L. sceleratus* is the most extensively studied species across all the considered categories and showed the most diverse reported or potential commercial uses (ranging from pharmaceutical to medical), while all other species were comparatively less represented in each category, as indicated in Figure 3. In this context, *P. miles* emerged as a peculiar case. It rapidly accumulated literature sources across categories, with a restricted time lag with respect to its explosion (three years), fueled by its status of iconic NIS status and influenced by the previous well-documented invasion of the *Pterois* spp. complex in the south-east Atlantic Ocean, which provided a global alert and a pre-existing framework to work on for Mediterranean researchers [73,74], allowing monitoring initiatives and research funding to be mobilized much faster than they would for less conspicuous or older colonizers. Ultimately, while *L. sceleratus* dominated the bibliometric landscape (46 publications across topics) due to its high perceived impact, the research trajectory of *P. miles* suggests that iconic invaders can bridge the knowledge gap much faster than less iconic or older colonizers.

The scarcity of data on fecundity and spawning periods for all species—regardless of the literature sources considered– required the collapse of these two categories into a “reproduction” category, highlighting the weakness in the current understanding of Lessepsian migrants’ reproduction within the invaded range. Indeed, for both literature datasets, most studies focused on length–weight relationships and diet, representing the most immediate data to collect with respect to long-term investigations required to collate quantitative information on species spawning periods, population growth and fecundity (Figure 3). Such scarcity in key biological traits is a generalized knowledge gap concerning many Mediterranean species, with reproductive traits being the least investigated [75,76]. Additionally, the vast majority of Mediterranean non-indigenous fish species have zero available biological records [76]. Finally, four of the five target species (*L. sceleratus*, *P. miles*, *S. luridus* and *S. rivulatus*) are considered the “well-studied” exceptions among Mediterranean NIS [76]. However, the lack of studies on their growth and reproduction highlights a critical knowledge gap persisting even for these prominent invaders. Although counterintuitive, this may be due to the perception of knowledge about a species. A pre-existing well-documented basis of information on a species may lead to the overlook of knowledge gaps; such bias may explain the lag in publications experienced by siganids and is highlighted in this study.

The exclusion of non-indexed sources from the database did not change the proportions of publications in each category across species, with the only exception of *L. sceleratus* due to the high contribution of non-indexed investigations on the species (over 50%). However, the exclusion of non-indexed sources affected the observed trends of literature accumulation (Figure 4), causing: (1) consistent lags across species and categories, as early studies were dominated by non-indexed sources; (2) slower accumulation rates across categories, in particular for the biological features and the commercial uses; (3) the absence of investigations on commercial uses in three species, as confirmed by keywords co-occurrence networks (Figure 5, Figure 6, Figure 7 and Figure 8); and (4) time-gaps in the literature accumulation, such as for *L. sceleratus* between 2013 and 2020. Such patterns are further confirmed by the collaboration networks and temporal trends in countries’ research effort (Figure 9). This confirmed that the exclusion of non-indexed sources can cause the introduction of linguistic and geographic bias: by including non-indexed sources, we were able to incorporate a high number of sources from North African and Levantine countries, including those in Turkish and French languages from Turkey and Lebanon. Excluding this regional literature not only creates a theoretical data gap, but it can directly impair specific management actions—most notably, early detection protocols and accurate range mapping—by omitting first records and regional expansion steps, ultimately flawing risk assessment procedures [22,23]. Addressing such gaps in the indexed knowledge is therefore essential to improve management strategies and modeling future spread of the species, ensuring that mitigation efforts are based on the full spectrum of available baseline data. However, we highlight the fact that the exclusion of sources in Arabic due to Google Scholar’s linguistic limitations might have caused further, unquantifiable biases in the results of our analyses. Therefore, further investigations should address the issue by including literature searches in Arabic-specific literature databases. Finally, from a closer inspection of the collaboration network (Figure 9), the Cyprus–UK, Greece–Sweden and Turkey–Canada links emerged as rooted in PhD and postgraduate research supervisions. This highlights that some international connections are driven mostly by individual academic mobilities rather than systemic bilateral cooperation between countries or well-established international research pipelines.

### 4.3. Study Limitations

While this study provides valuable insights, several methodological limitations must be acknowledged when interpreting its findings. First, our methodology relied on extensive literature searches in Google Scholar. However, unlike traditional IBD, the Google Scholar search algorithm is more dynamic and produces fluctuating results, complicating the exact replication of our search queries [77,78]. Furthermore, caution is required when interpreting the cumulative curves of publication growth. Although frequently used in bibliometric analyses, they only serve as an indirect proxy of scientific attention and do not reflect actual paradigm shifts and methodological quality of the sources [79]. Finally, the key finding of our investigation was that non-indexed literature contributes substantial and relevant information to research on bioinvasions. However, because non-indexed literature also includes non-peer-reviewed gray literature, a critical trade-off emerges between the inclusiveness and the source quality. Since gray literature encompasses a broad spectrum of documents—including reports, preprints, doctoral theses, conference abstracts and posters—its quality, reliability and rigor can vary significantly [80]. While inclusion of gray literature allows the minimization of any publication bias—thus offering a more comprehensive overview of bioinvasions—it can also introduce noise and heterogeneous data quality that must be carefully weighted during evidence synthesis.

## 5. Conclusions

Lessepsian invasions are currently reshaping ecosystems, posing increasing ecological, economic and public health threats. Thus, the development of effective, evidence-based management strategies has become a priority. In this context, by focusing on five representative Lessepsian fish species, the present study provided an updated overview of the invasion dynamics, distribution patterns, and evolution of scientific research across the basin, emphasizing the importance of including non-indexed sources to improve the robustness of the reconstruction, and their exclusion introduced temporal, geographic biases and linguistic barriers in the analysis of bibliometric patterns. Different invasion dynamics were observed for the five species, with *P. miles* and *Siganus* spp. exhibiting a current exponential expansion pattern and *F. commersonii* and *L. sceleratus* showing signs of expansion saturation. Among all species, *L. sceleratus* displayed the shortest arrival phase and fastest proliferation, likely resulting from lags in species detection and the high-impact profile of the species. Bibliometric analyses revealed that research effort is not always proportional to spatial expansion. In particular, *L. sceleratus* received disproportionate attention because of its strong economic and public health implications, thus becoming the most extensively studied species across thematic categories. Conversely, the rapid development of the literature on *P. miles* highlights how prior invasion events in other global regions (i.e., the Caribbean) and the status of a globally iconic invader can accelerate the response of the scientific community in newly invaded regions. Finally, we highlighted important knowledge gaps in reproductive biology and population dynamics of Lessepsian fishes in the Mediterranean. Addressing such gaps will be essential to improve management strategies and modeling future spread of the species.

## Figures and Tables

**Table 1:** Number of total Mediterranean occurrences, number and proportion of occurrence records for the source Biodiversity Information Facilities, Indexed Bibliographic Databases and Non-Indexed Bibliography (BIFs, IBDs, NIB, respectively), and date and country of first Mediterranean occurrence for each species included in the analysis.

Species	No. of Occurrences	BIFs	IBDs	NIB	First Occurrence (Year; Country)
*Fistularia commersonii*	976	53.5% (522)	32.7% (319)	13.8% (135)	1975; Lebanon
*Lagocephalus sceleratus*	759	54% (410)	34.7% (263)	11.3% (86)	2003; Turkey
*Pterois miles*	1828	48.9% (893)	46.7% (853)	4.5% (82)	1991; Israel
*Siganus luridus*	2099	53.8% (1130)	41.7% (875)	4.5% (94)	1932; Greece
*Siganus rivulatus*	1678	52.7% (886)	44.1% (742)	3.2% (53)	1924; Palestine

**Table 2:** Output of the segmented and linear models for the three segments, including the breakpoints (BP1 and BP2) and their confidence intervals (within square brackets), the final slopes of each segment, and the t- and *p*-values associated with each slope variation (Slope 1, Slope 2, Slope 3), and the global adjusted R^2^.

Species	Variable	BP 1	BP 2	T50	Slope 1	Slope 2	Slope 3	R^2^ Adj
*Fistularia commersonii*	Occupied Cells	2002.53 [2002.18–2002.88]	2020.51 [2019.19–2021.83]	2013	0.25 t = 1.57 *p* = 0.124	23.88 t = 67.46 *p* < 0.001	13.2 t = −4.88 *p* < 0.001	0.998
	Publications	1998.58 [1994.22–2002.95]	2004.51 [2003.52–2005.50]	2015	0t = 0 *p* = 1	4.07 t = 1.74 *p* = 0.089	22.61 t = 7.87 *p* < 0.001	0.996
*Lagocephalus sceleratus*	Occupied Cells	2005.57 [2004.45–2006.69]	2019.62 [2018.75–2020.48]	2013	4.5 t = 0.7 *p* = 0.495	27.92 t = 3.61 *p* = 0.002	6.37 t = −9.51 *p* < 0.001	0.997
	Publications	2008.38 [2005.59–2011.18]	2012.89 [2011.16–2014.62]	2018	0t = 0 *p* = 1	12.27 t = 1.99 *p* = 0.063	31.47 t = 3.49 *p* = 0.003	0.994
*Pterois miles*	Occupied Cells	2011.23 [1993.76–2028.70]	2013.76 [2012.13–2015.39]	2019	0.24 t = 0.32 *p* = 0.751	5 t = 0.16 *p* = 0.871	56.52 t = 1.77 *p* = 0.087	0.990
	Publications	1992.86 [NaN–NaN]	2017.68 [2017.30–2018.06]	2022	0t = 0 *p* = 1	0t = 0 *p* = 1	82.62 t = 27.29 *p* < 0.001	0.986
*Siganus luridus*	Occupied Cells	2001.4 [2000.36–2002.44]	2013.5 [2012.28–2014.72]	2016	0.82 t = 10.19 *p* < 0.001	17.17 t = 14.41 *p* < 0.001	35.27 t = 11.3 *p* < 0.001	0.993
	Publications	1984.97 [1980.31–1989.62]	1997.09 [1993.03–2001.16]	2007	0t = 0 *p* = 1	8.84 t = 3.4 *p* = 0.001	20 t = 4.12 *p* < 0.001	0.970
*Siganus rivulatus*	Occupied Cells	1997.31 [1995.06–1999.56]	2014.40 [2013.78–2015.02]	2019	0.51 t = 6.49 *p* < 0.001	7.45 t = 9.65 *p* < 0.001	43.34 t = 23.14 *p* < 0.001	0.988
	Publications	1990.41 [1989.47–1991.36]	2010.55 [2007.08–2014.02]	2005	0.49 t = 4.84 *p* < 0.001	17.66 t = 28.37 *p* < 0.001	11.41 t = −5.49 *p* < 0.001	0.992

**Table 3:** Proportion of literature sources indexed (IBD) and not indexed (NIB) in WoS and Scopus databases.

Species	IBD Sources	NIB Sources
*Fistularia commersonii*	62.79%	37.21%
*Lagocephalus sceleratus*	43.42%	56.58%
*Pterois miles*	74.19%	25.81%
*Siganus luridus*	66.67%	33.33%
*Siganus rivulatus*	77.27%	22.73%

**Table 4:** Delta T50 (ΔT50) for the two curves and results of GLS models, including the β estimate and its standard error, its *p*-value, the autoregressive parameters Φ for the GLS-AR1 models, and the log-likelihood of the model.

Species	ΔT50	β (±SE)	*p*-Value	φ (AR1)	Log Likelihood
*Fistularia commersonii*	2	0.53 ± 0.13	<0.001	0.13	−63.96
*Lagocephalus sceleratus*	5	−0.06 ± 0.22	0.78	0.35	−32.08
*Pterois miles*	3	0.89 ± 0.18	<0.001	0.47	−36.65
*Siganus luridus*	−9	0.14 ± 0.12	0.25	0.36	−126.95
*Siganus rivulatus*	−14	0.59 ± 0.11	<0.001	0.41	−127.78

**Table 5:** Number of nodes and density of computed networks.

	Total Sources		Indexed Sources	
Species	Nodes Number	Density	Nodes Number	Density
*Fistularia commersonii*	53	0.11	40	0.15
*Lagocephalus sceleratus*	76	0.07	53	0.09
*Pterois miles*	53	0.12	44	0.16
*Siganus rivulatus*	96	0.10	87	0.12

## Data Availability

The database compiled for this study is part of the ongoing VALIAS project. To comply with project-specific data sharing policies, the complete dataset and associated metadata will be made publicly available in an open access repository upon the formal conclusion of the VALIAS project.

## Abbreviations

The following abbreviations are used in this manuscript:

IBD	International Bibliographic Database
BIFs	Biodiversity Information Facilities
NIB	Non-Indexed Bibliographic
NIS	Non-Indigenous Species
IAS	Invasive Alien Species
GES	Good Environmental Status
MSFD	Marine Strategy Framework Directive
FAO	Food and Agriculture Organization
GFCM	General Fishery Commission for the Mediterranean
GBIF	G Biodiversity Information Facility
OBIS	Ocean Biodiversity Information System
EEA	European Environmental Agency
BP	Breakpoint
GLS	Generalized Least Square
AR1	First-order Autoregressive Correlation Structure
REML	Restricted Maximum Likelihood
WoS	Web of Science
LWR	Length–Weight Relationship

## Appendix A. Detailed Description of the Search Procedure Used to Collate the Literature Sources

The queries for each species were structured as follows:

The first part of the query was dedicated to the names of the species, both scientific and common names. All the common names utilized for the species were:

1. *Fistularia commersonii*: “bluespotted cornetfish”;
2. *Lagocephalus sceleratus*: “silver-cheeked toadfish”, “silverstripe blaasop”;
3. *Pterois miles*: “lionfish”, “devil firefish”;
4. *Siganus luridus*: “dusky spinefoot”;
5. *Siganus rivulatus*: “marbled spinefoot”.

In addition to this, a second block was added for specifically checking on the invaded range, structured as (“Mediterranean” OR “invaded range”). Finally, a third block was added, containing the specific topics we focused on:

1. Occurrence: “occurrence”, “first record”, “new record”;
2. LWR: “LWR”, “length–weight relation”, “length–weight relationship”;
3. Growth: “growth parameters”, “von Bertalanffy”, “size at maturity”, “population dynamics”;
4. Reproduction: “spawning”, “fecundity”, “reproduction”, “reproductive season”;
5. Trophic ecology: “stomach content”, “diet composition”, “food items”, “feed habits”, “trophic ecology”;
6. Uses: “pharmaceutic”, “cosmetics”, “medical use”, “commercial use”, “valorization”, “human consumption”, “chemical composition”.

**Table A1:** Table of the number of total retrieved sources and number of retained papers (in parentheses). The last column regards the final number of papers on the species after the records have been screened and de-duplicated. LWR = length–weight relationships.

Species	Occurrence	LWR	Growth	Reproduction	Trophic Ecology	Uses	Final Retained Papers
*F. commersonii*	933 (91)	117 (23)	233 (7)	674 (4)	207 (9)	112 (3)	107
*L. sceleratus*	1170 (88)	196 (31)	302 (16)	867 (11)	221 (10)	320 (10)	112
*P. miles*	2410 (58)	175 (9)	988 (5)	2440 (4)	772 (9)	625 (6)	83
*S. luridus*	1140 (91)	154 (17)	336 (5)	891 (4)	289 (9)	180 (1)	111
*S. rivulatus*	1270 (67)	267 (25)	512 (7)	1300 (6)	295 (11)	441 (1)	95
						**TOT**	366

Literature records were included if they met the following inclusion criteria:

(1) Focused on at least one of the five target alien fish species (*F. commersonii*, *L. sceleratus*, *P. miles*, *S. luridus*, *S. rivulatus*);
(2) Contained primary data or verified records regarding the Mediterranean Sea (invaded range);
(3) Provided quantitative or qualitative information on at least one of the selected categories (occurrence, LWR, growth, reproduction, trophic ecology, or uses).

Conversely, papers were excluded based on the following explicit exclusion criteria:

(1) Literature records not including information from the Mediterranean Sea or not focusing on the target species;
(2) Literature records with no available text;
(3) Literature records with insufficient, ambiguous, or missing data;
(4) Redundant duplicate reports.

We specify that most of the literature records initially retrieved by Google Scholar included studies out of the Mediterranean (especially for *P. miles*) or did not contain specific information on the target species (due to keyword ambiguity). All the literature meeting these two primary pre-requisites was thoroughly screened to allow the recording of occurrence information, when available, and subsequently analyzed for the other biological and socio-economic topics. All the duplicate records were initially removed by human inspection and later on checked in R with the use of the *bibliometrix* package.
